# Why Attending ABRF Conferences are Important to Me

**Published:** 2026-03-06

**Authors:** Ron Orlando

**Affiliations:** 1 ccrc University of Georgia

**Keywords:** test, ABRF Conference

## Abstract

When asked why attending annual ABRF conferences is important to me, the answer is easy. I may leave the conference with an idea or learn something – of course, I can do these things in my office, reading papers and trying experiments in the lab. Connections with other people – I cannot do this at “home”. Without these other people, those synergistic discussions don’t occur. I have also realized that science is expanding faster than I can keep up. It’s a good thing that I don’t need to… It’s not what you know, but who you know. I don’t need to keep up; I have lots of friends, thanks to ABRF conferences, who stay abreast of everything in their areas, and I can tap into their expertise whenever I need to.

As a young graduate student, I attended a conference that remains pivotal in my career. Why, you ask? It wasn’t the science discussed, which I’ve long forgotten, but a conversation I had with a fellow attendee. His words still resonate with me. He said, “It’s not what you know, it’s who you know that matters.” When I asked, he explained that knowing an expert meant I could simply ask them rather than mastering the subject myself. I immediately saw the wisdom. At the time, I was a mass spectrometrist unfamiliar with liquid chromatography, even though LC-MS was widely used. I saw LC as just a way to introduce samples into the MS. A few years later, at another conference, I met Andy Alpert, a separation scientist who became my go-to LC expert. He was happy to share his knowledge whenever I needed. Andy had invented hydrophilic interaction chromatography (HILIC). One of our discussions led us to realize that HILIC would be excellent for carbohydrate separation. Our collaboration had far-reaching consequences. On a personal level, my career was enhanced by demonstrating for the first time that isomeric glycans could be separated by HILIC-MS. Over thirty years later, HILIC is our go-to separation strategy. This new analytical strategy helped us better support the researchers who utilize my laboratory. The scientific community as a whole benefited with the analysis of glycans by HILIC-MS now a common approach; in 2025 alone, almost 9,000 papers used HILIC for glycan analysis. This is just one example of how I have benefited from attending ABRF conferences.

Thanks to my attendance at numerous ABRF conferences, I have a large network of colleagues (many of whom I now call friends). I can call them (and they will answer or at least return the call), and thus I do not need to know about DNA/RNA sequencing, metagenomics, antibody development/validation, imaging, and the list goes on. And they do not need to know anything about characterizing glycoproteins because they can (and do) call me.

When asked why attending annual ABRF conferences is important to me, the answer is easy. I may leave the conference with an idea or learn something – of course, I can do these things in my office, reading papers and trying experiments in the lab. But, when it comes to connections with other people, I cannot do this at “home”. Without these other people, those synergistic discussions don’t occur. I have also realized that science is expanding faster than I can keep up. It’s a good thing that I don’t need to: It’s not what you know, but who you know. I don’t need to keep up; I have lots of friends, thanks to ABRF conferences, who stay abreast of everything in their areas, and I can tap into their expertise whenever needed.

